# Subcellular Localization of Seed-Expressed LEA_4 Proteins Reveals Liquid-Liquid Phase Separation for LEA9 and for LEA48 Homo- and LEA42-LEA48 Heterodimers

**DOI:** 10.3390/biom11121770

**Published:** 2021-11-25

**Authors:** Orarat Ginsawaeng, Carolin Heise, Rohit Sangwan, Daniel Karcher, Itzell Euridice Hernández-Sánchez, Arun Sampathkumar, Ellen Zuther

**Affiliations:** Max Planck Institute of Molecular Plant Physiology, Am Mühlenberg 1, 14476 Potsdam, Germany; ginsawaeng@mpimp-golm.mpg.de (O.G.); heise_carolin@web.de (C.H.); rohit.sangwan@uni-potsdam.de (R.S.); karcher@mpimp-golm.mpg.de (D.K.); itzellbiologiam@gmail.com (I.E.H.-S.); sampathkumar@mpimp-golm.mpg.de (A.S.)

**Keywords:** subcellular localization, LEA_4 proteins, seed-expressed, cytoplasmic condensates, heterodimer

## Abstract

LEA proteins are involved in plant stress tolerance. In Arabidopsis, the LEA_4 Pfam group is the biggest group with the majority of its members being expressed in dry seeds. To assess subcellular localization in vivo, we investigated 11 seed-expressed LEA_4 proteins in embryos dissected from dry seeds expressing LEA_4 fusion proteins under its native promoters with the Venus fluorescent protein (*proLEA_4::LEA_4:Venus*). LEA_4 proteins were shown to be localized in the endoplasmic reticulum, nucleus, mitochondria, and plastids. LEA9, in addition to the nucleus, was also found in cytoplasmic condensates in dry seeds dependent on cellular hydration level. Most investigated LEA_4 proteins were detected in 4-d-old seedlings. In addition, we assessed bioinformatic tools for predicting subcellular localization and promoter motifs of 11 seed-expressed LEA_4 proteins. Ratiometric bimolecular fluorescence complementation assays showed that LEA7, LEA29, and LEA48 form homodimers while heterodimers were formed between LEA7-LEA29 and LEA42-LEA48 in tobacco leaves. Interestingly, LEA48 homodimers and LEA42-LEA48 heterodimers formed droplets structures with liquid-like behavior. These structures, along with LEA9 cytoplasmic condensates, may have been formed through liquid-liquid phase separation. These findings suggest possible important roles of LLPS for LEA protein functions.

## 1. Introduction

Late embryogenesis abundant (LEA) proteins were first described 40 years ago [1,2,3]. LEA proteins were named “late embryogenesis proteins” as they were originally found in that developmental stage; however, they are also expressed in other plant organs. According to their shared physicochemical properties, LEA proteins were assigned to subgroups. LEA proteins from two of these subgroups and LEA-like proteins are also expressed in other organisms including bacteria and invertebrates [4,5,6,7,8].

LEA proteins are generally small and hydrophilic molecules, containing high proportions of Gly, Ala, Glu, Lys/Arg, and Thr while their Cys and Trp contents are low or absent [9]. With limited hydrophobic residues, most LEA proteins lack conventional secondary structure and are not susceptible to protein aggregation even upon boiling [10,11]. This also means that most LEA proteins are naturally unfolded in a fully hydrated environment. Thus, the majority of LEA proteins are classified as intrinsically disordered proteins (IDP) [12,13,14]. Interestingly, LEA proteins reversibly gain secondary structure, predominantly alpha-helix, in a partially or completely dehydrated environment triggered by desiccation or freezing and/or high molecular crowding [9,13]. LEA proteins were suggested to be able to perform their function without acquiring structures [15]. On the other hand, as multifunctional proteins, LEA proteins may sense physical and chemical changes in their surroundings, for example by phosphorylation or dephosphorylation, which may mediate changes in their protein conformation and allow LEA proteins to have specific interactions and functions depending on the degree of protein folding [12,14].

It is widely known that LEA proteins are involved in plants’ responses to abiotic stresses that evoke cellular dehydration, including salt, drought, and cold stress [9,12]. In this regard, the expression of LEA genes is highly induced in *Arabidopsis thaliana* mature leaves when plants are exposed to these stresses [16]. The fact that LEA proteins are highly abundant in dry mature seeds, resurrection plants, and many anhydrobiotic organisms further suggests their roles in preserving cellular viability during extreme dehydration [5]. Despite this, the protective mechanisms of LEA proteins are still inconclusive in vivo. In vitro evidence suggested that LEA proteins ensure enzyme activity, act as a molecular shield preventing protein aggregation and as chaperone, stabilize and associate with membranes, sequester ions and reactive oxygenic species, prevent cellular water loss by acting as hydration buffer, and stabilize sugar glasses by increasing the glass transition temperature [4,5,12,14,17]. In addition, the IDP-structure of LEA proteins might contribute to a phenomenon named liquid-liquid phase separation (LLPS), a process which gives rise to proteinaceous membrane-less organelles [18]. Nevertheless, not all IDPs undergo LLPS and intrinsically disordered regions (IDRs) are not necessarily required for LLPS.

LEA proteins are able to interact with each other or other proteins. Direct LEA-target protein interactions have been shown among the LEA_2, dehydrin, LEA_4, and the SMP protein families [18]. As an example, dehydrins AtCOR47, AtERD10, and AtRAB18 bound to the aquaporin AtPIP2B and a homodimeric interaction of RAB18 complexes was revealed as well as a heterodimeric association between AtRAB18 acidic dehydrins [19]. Moreover, associates of COR15A with the small and large subunit of Rubisco, potentially prevented a freezing induced deactivation of the enzyme [18]. Two other dehydrins from Arabidopsis, ERD14 (At1g76180), and HIRD11 (At1g54410) interacted with the Arabidopsis Phi9 Glutathione-S-Transferase9 and the Arabidopsis leucine-rich repeat receptor-like kinase (LRR-RLK) Phloem Intercalated with Xylem-Like 1 (AtPXL1), respectively [18].

In *Arabidopsis thaliana*, there are a total of 51 LEA proteins organized across nine different groups [16]. Among these groups, Pfam group LEA_4 (also known in other nomenclatures as group 3/group 5 [20] and D-7/D-29 [21]) is the largest group with 18 members with the majority of them highly expressed in dry mature seeds. One of the interesting features of the LEA_4 Pfam group is the high occurrence in non-plant models [5], suggesting that the LEA_4 group is conserved through evolution. Noteworthy is also the high diversity among LEA_4 members in terms of polypeptide size, sequence, and degree of hydrophilicity [16].

Subcellular localization is an important information required to unravel protein functions. Although computational approaches provide interesting insights, high accuracy is not always achieved and so it does not eliminate the importance of in vivo investigations. It has been shown before that LEA proteins localize in various subcellular components both in vivo [22,23,24,25,26,27,28,29,30,31]; and in silico [6,16,23,24,25,30,32]. In Arabidopsis, subcellular localizations of 51 LEA proteins were identified in protoplasts and some also in plants and seedlings [30]. Nevertheless, the subcellular localization of LEA proteins in dry seeds was not previously investigated.

In this study, bioinformatics approaches were used to assess subcellular localization of 11 seed-expressed LEA_4 and their promoter motifs. Subcellular localization studies on 11 seed-expressed LEA_4 fusion proteins under their native promoters were conducted in embryos of Arabidopsis dissected from dry mature seeds. Strikingly, we found cytoplasmic condensates of LEA9 in embryos under different conditions. The study was further expanded to examine the expression of 11 seed-expressed LEA_4 proteins in plant organs in young seedlings. Based on our subcellular localization study in dry seeds, we investigated homo- and heterodimeric interactions of closely related LEA proteins that localize in the same subcellular compartment. Furthermore, LLPS behavior was described for specific LEA_4 proteins and protein interactor pairs in vivo.

## 2. Materials and Methods

### 2.1. Bioinformatic Analysis of Seed-Expressed LEA_4 Proteins

Protein sequences of 11 seed-expressed LEA_4 proteins (LEA7, LEA9, LEA19, LEA25, LEA28, LEA29, LEA30, LEA36, LEA42, LEA43, and LEA48 [16]) were retrieved from their representative gene model in the Arabidopsis Information Resource (TAIR) database (https://www.arabidopsis.org (accessed on 29 April 2021)). The following online tools for subcellular localization prediction were used: BUSCA [33], DeepLoc-1.0 [34], LOCALIZER [35], LocTree3 [36], Plant-mSubP [37], pLoc-mPlant [38], and TargetP-2.0 [39]. The disorder content for some of the protein sequences was predicted using the MFDp2 prediction tool [40]. For LEA9, the ELM prediction tool was additionally used to search for SLiMs in its protein sequence [41]. AGRIS AtcisDB database (https://agris-knowledgebase.org/AtcisDB/ (accessed on 24 May 2021)) was used to search for promoter motifs in the intergenic sequences (maximum of 3 kb upstream) of seed-expressed LEA_4 genes [42]. The frequency of each promoter motif was summarized in Supplementary Table S1A. Common promoter motifs of LEA proteins in the same subcellular structure were analyzed using “VennDiagram” package in R studio version 1.4.1106 [43] and are shown in Supplementary Table S1B.

### 2.2. Construction of Transgenic Lines Containing Seed-Expressed proLEA_4::LEA_4:Venus Constructs

Plasmid vector containing full length Venus [44] and genomic DNA of *A. thaliana* Col-0 accession with native promoters and introns (Supplementary Table S2) were used as template to amplify full length sequences of Venus and 11 seed-expressed LEA_4 genes using Phusion^®^ High-Fidelity DNA Polymerase (New England BioLabs, Ipswich, MA, USA). For used primers, see Supplementary Table S3. PCR products were purified from agarose gel using NucleoSpin^®^ Gel and PCR Clean-up kit (Macherey-Nagel, Düren, Germany) and cloned into binary vector pORE-O2 [45] using In-Fusion^®^ HD Cloning kit (Takara Bio, Kusatsu, Japan). Venus was cloned into *Sal*I and *Spe*I sites and each LEA_4 gene sequence into *Sma*I site upstream of Venus. The In-Fusion reactions were transformed into 100 µL TOP10 chemically competent *E. coli* using 42 °C for 45 s. Transformed *E. coli* cells were grown on LB plates containing kanamycin (50 µg/mL) either at 37 °C overnight or at 28 °C for two days. Colonies were selected for isolation of plasmids using modified alkaline-lysis method [46]. LEA_4 and Venus sequences for each construct were confirmed by sequencing (LGC Genomics GmbH). The constructs were transformed into electrocompetent *Agrobacterium tumefaciens* strain GV3101 using 1800 V. Transformed Agrobacterium was grown on YEB plates containing kanamycin (50 µg/mL) at 28 °C for two days. Positive colonies were identified by PCR using isolated plasmids as templates [46] and LEA_4 gene- and vector-specific primers. Positive clones were inoculated in liquid YEB with kanamycin (50 µg/mL) at 28 °C for one day followed by plating on YEB plates containing kanamycin (50 µg/mL), gentamycin (25 µg/mL), and rifampicin (100 µg/mL). Agrobacterium with plasmids of interest were transformed into *A. thaliana* Col-0 accession by floral dipping [47]. Seeds from T0 and T1 generations for each construct were sterilized using 70% EtOH with 0.001% triton for 5 min and absolute EtOH for 5 min, followed by stratification in the dark at 4 °C for two days on MS plates containing kanamycin (50 µg/mL) and 1% sucrose. MS plates were transferred to growth chamber at 22 °C for two weeks. Seedlings were then transferred to soil and grown in a greenhouse (16 h light period, 21 °C/19 °C, and 50%/50% relative humidity (RH) day/night) until seed harvest. Mature siliques were bagged and dried in the greenhouse for two weeks before seeds were collected at 15 °C and 15% RH for at least three weeks.

### 2.3. Subcellular Localization of Seed-Expressed LEA_4 Proteins in Embryos

Embryos dissected from imbibed T2 seeds of two to three independent *proLEA_4::LEA_4:Venus* transgenic lines were inspected under a confocal microscope producing 6 to 12 images across embryos per line before further investigation to confirm consistency in subcellular localization pattern. A transgenic line of each construct was either crossed with marker lines to verify ER and plastids localization or the embryos were stained to verify nucleus and mitochondria localization. Verification of LEA proteins in ER and plastids was done by crossing *proLEA_4::LEA_4:Venus* T2 generation with ER marker line ER-ck or plastid marker line Pt-ck expressing cyan fluorescent protein (CFP) in the respective organelle [48] ordered from Nottingham Arabidopsis Stock Centre (NASC). Seeds from crossings were imbibed in water for approximately 1 h before embryos were dissected for imaging. Verification of LEA proteins in the nucleus and mitochondria was carried out using either 10 µM DAPI (Sigma-Aldrich, St. Louis, MO, USA) or 10 nM Mitotracker Orange CMRMRos staining for 5–10 min (Invitrogen, Waltham, MA, USA) in embryos dissected from T3 seeds imbibed in water overnight, except for LEA9 where the seeds were imbibed for 1 h before staining with the MitoTracker. From crossings with organelle marker lines and embryos dyed with organelle-specific dyes, 3 to 5 embryos from one line were investigated with 15 to 30 images and 8 to 12 images, respectively, from cotyledons, showing all the reported localization pattern. Observations were carried out under a confocal microscope SP8 (Leica, Wetzlar, Germany) using a 20× objective lens. All images in this study were processed with the software FIJI [49].

### 2.4. Investigation of Cytoplasmic Condensates of LEA9

T3 seeds of *proLEA9::LEA9:Venus* transgenic lines were imbibed in the following conditions before the embryos were dissected out for confocal microscope observation: water (for 1 and 24 h), glycerol (for 1 and 24 h), 1 mg/mL cycloheximide (for 1 h), and 2 M NaCl (for 1 h). The observation was carried out (*n* = 3–5) under confocal microscope SP8 (Leica, Wetzlar, Germany) using a 20× objective lens. LEA9 condensates in embryos dissected from seeds in water or cycloheximide were quantified as counts/area (µm^−2^) based on 3 to 5 embryos per conditions (*n* = 9).

### 2.5. Expression of Seed-Expressed LEA_4 Proteins in Seedlings

T3 seeds of *proLEA_4::LEA_4:Venus* transgenic lines were sterilized, stratified and grown on MS plates as mentioned above. Four-day-old seedlings from all lines were transferred to liquid MS with 1% sugar for 4–5 h (60 rpm) before observation (*n* = 3). Expression of LEA proteins was investigated under confocal microscope SP8 (Leica, Wetzlar, Germany) using 5× and 20× objective lens.

### 2.6. Construction of Expression Vectors for Seed-Expressed LEA_4

Gateway method was used for cloning. Open reading frames (ORF) of *LEA7*, *LEA28*, *LEA29*, *LEA42*, and *LEA48* were amplified using Phusion^®^ High-Fidelity DNA Polymerase (New England BioLabs, Ipswich, MA, USA) with appropriate *att*B primers and DNA extracted from its respective *proLEA_4::LEA_4:Venus* transgenic lines as templates. For primers see Supplementary Table S3. The PCR products were purified from agarose gel using NucleoSpin^®^ Gel and PCR Clean-up kit (Macherey-Nagel, Düren, Germany). BP reactions of *att*B-*LEA*-PCR products were carried out with either donor vector pDONR™221 P1-P4 or pDONR™221 P3-P2 using BP Clonase™ II enzyme mix (Invitrogen, Waltham, MA, USA). BP reactions were dialyzed against ddH2O on a 0.025 µm Millipore filter membrane for 30–45 min followed by transformation into *E. coli* TOP10 cells using electroporation method. The transformed *E. coli* were plated on LB medium with kanamycin (50 µg/mL) and incubated overnight at 37 °C. Colony PCR using AccuStart™ II SuperMix (2×) (Quantabio, Beverly, MA, USA) from liquid overnight cultures was performed to confirm positive clones and positive plasmids were isolated using the NucleoSpin^®^ Plasmid/Plasmid (NoLid) Kit (Macherey-Nagel, Düren, Germany). LR reactions of entry vectors and destination vector pBiFCt-2in1-CC were performed using LR Clonase™ II enzyme mix (Invitrogen, Waltham, MA, USA). LEA genes cloned only at *att*R1- R4 site of the destination vector acted as negative controls for confocal microscope studies. The entry vectors were linearized with *EcoN*I to facilitate the recombination of LR reactions. After dialysis and transformation as carried out for BP reactions, the *E.coli* cells containing the expression vector were streaked on LB medium plates containing spectinomycin (120 µg/mL), IPTG (0.1 mM), and X-Gal (40 µg/mL) for blue-white selection after overnight incubation at 37 °C. Positive colonies were confirmed by colony PCR as described above and by sequencing of plasmids (LGC Genomics GmbH) isolated using the NucleoSpin^®^ Plasmid/Plasmid (NoLid) Kit (Macherey-Nagel, Düren, Germany).

### 2.7. Homo- and Heterodimer Complex Formation of Seed-Expressed LEA_4 Proteins in Tobacco Leaves

The expression vectors were transformed into A. tumefaciens strain GV3101 using 1800 V. Colonies were subsequently selected on LB plates containing spectinomycin (120 µg/mL), gentamycin (30 µg/mL), and rifampicin (100 µg/mL) for two days at 28 °C and confirmed to be positive using colony PCR. Positive clones were inoculated in liquid LB media containing the same antibiotics and under the same growth condition. In parallel, liquid LB media (kanamycin (50 µg/mL), gentamycin (30 µg/mL), and rifampicin (100 µg/mL)) was inoculated with Agrobacteria carrying the expression vector of the p19 protein, which avoids gene silencing [50]. After the cultures were refreshed and incubated overnight, 5 mL of liquid LB media of the inoculated Agrobacteria containing the expression vector of interest were mixed in a ratio 1:1 with the liquid LB media inoculated with Agrobacteria carrying the expression vector of the p19 protein. The mixture was infiltrated on the abaxial side of 4-week-old Nicotiana benthamiana leaves. The plants were left on wet paper towels at room temperature in the dark for 4 d without watering before observation. Immunofluorescence images were recorded with SP5 confocal light microscope (Leica, Wetzlar, Germany) using a 20× objective lens. The formation of the homo- and heterodimeric complexes was assessed using the relative YFP/RFP fluorescence ratio and cut-off above 80% (n = 15–25).

To assess LLPS of LEA48 homodimers and LEA42-LEA48 heterodimers in tobacco leaves, leaf samples were incubated for 1 h in 10% 1,6-hexanediol (Sigma-Aldrich, St. Louis, MO, USA) and vacuum-infiltrated for 10 min before visualization (n = 4–5). For control, 1,6-hexanediol solution was substituted with water.

## 3. Results

### 3.1. Localization Prediction of Seed-Expressed LEA_4 Proteins Is Varied

Previously predicted LEA proteins localization using numerous algorithms were mostly non-unanimous [30]. To observe the performance of other newer released bioinformatic tools, six tools were selected to predict the localization of 11 seed-expressed LEA_4 proteins (LEA7, LEA9, LEA19, LEA25, LEA28, LEA29, LEA30, LEA36, LEA42, LEA43, and LEA48) [16] as follows: BUSCA [33], DeepLoc-1.0 [34], LOCALIZER [35], LocTree3 [36], Plant-mSubP [37], pLoc-mPlant [38], and TargetP-2.0 [39]. The most frequently predicted sites among 11 seed-expressed LEA_4 proteins were cytoplasm and nucleus (Supplementary Table S4). Regardless, the prediction results were partly inconclusive. These results stressed once again the difficulties in predicting subcellular localization of LEA proteins.

### 3.2. Differential Subcellular Localization of LEA_4 Proteins Expressed in Embryos from Dry Seeds

Observation of subcellular localization of LEA proteins was previously carried out in plants, seedlings and/or protoplasts using the 35S promoter [30]. We selected 11 LEA proteins from the LEA_4 Pfam group known to be seed-expressed [16] for the investigation of their localization using their native promoters. Native promoters along with full length genomic DNA of each LEA protein were fused at the carboxy ends with the Venus fluorescent protein [44] and transformed into *Arabidopsis thaliana*. Embryos dissected from hydrated mature seeds of a transgenic lines per construct were subjected to confocal laser scanning microscopy (CLSM) (*n* = 3–5). The same pattern of protein expression was found in cotyledons and embryonic axis for all investigated LEA proteins.

The subcellular localization of seed-expressed LEA_4 in embryos under their native promoters was widely diverse, some of them exhibiting single and dual subcellular localizations (Table 1. The most common subcellular structure observed resembled cytoplasmic strands and endoplasmic reticulum (ER).

**Figure 1:**
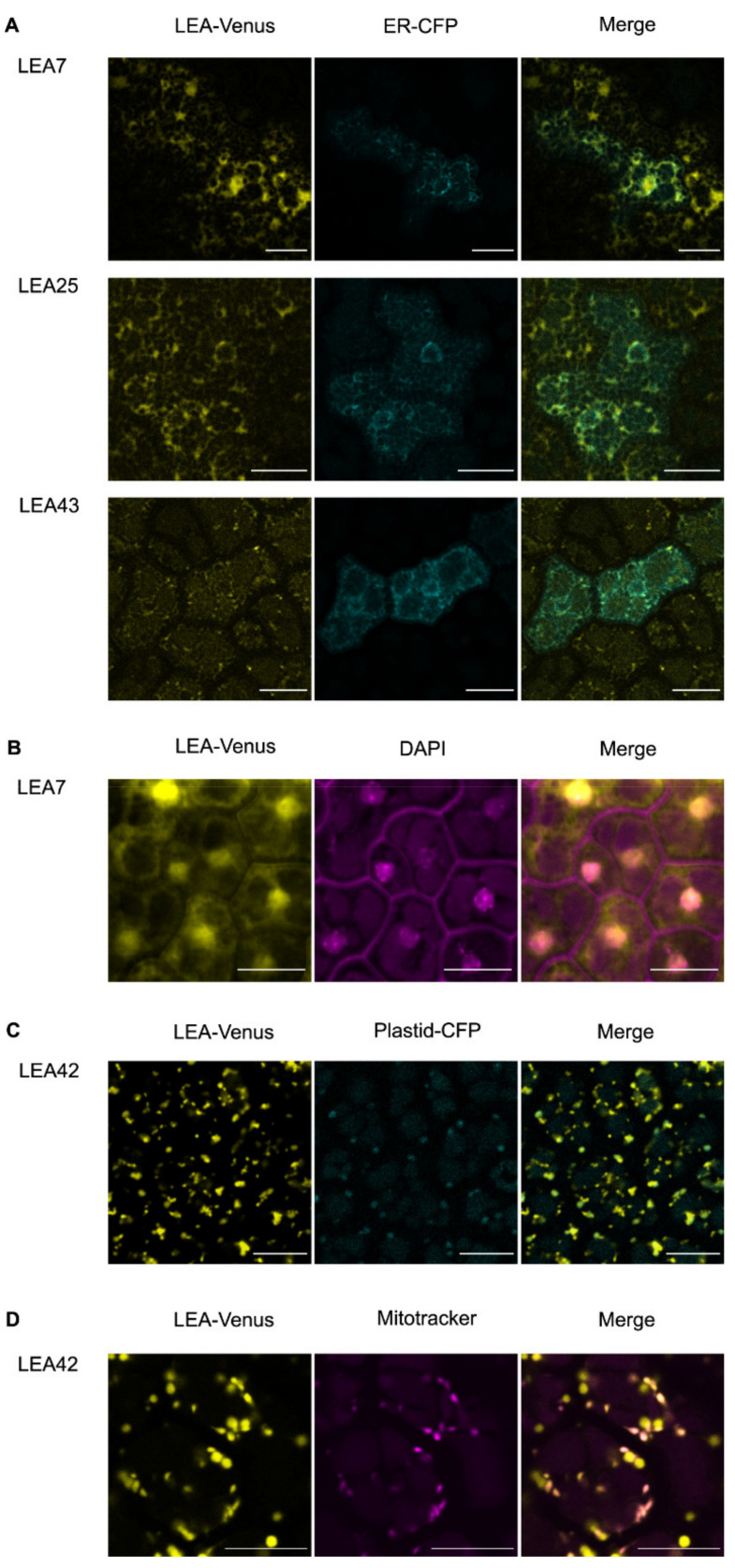
Subcellular localization of LEA7, LEA25, LEA42, and LEA43 in (**A**) ER, (**B**) nucleus, (**C**) plastids, and (**D**) mitochondria in embryos of dry seeds. *proLEA_4::LEA_4:Venus* lines were either crossed with an organelle marker line with CFP fluorescence (ER-ck or Pt-ck) or the dissected embryos were stained using organelle-specific dyes (DAPI or Mitotracker). Bars indicate 10 µm. Additional LEA proteins with similar localization are shown in Supplementary Figures S1–S3.

To assess this, we crossed the LEA translational reporter lines with plants expressing the fluorescent Cyan protein fused to an ER protein marker [48]. The majority of the expressed LEA proteins (LEA7, LEA19, LEA25, LEA28, LEA29, LEA30, LEA36, and LEA43) overlapped with ER structures observed in the ER reporter line (Figure 1A; Supplementary Figure S1A). However, it should be noticed that diffused cytoplasmic signals were detected for these LEA proteins, so that a cytoplasmic localization cannot be completely excluded.

Interestingly, by using time-lapse confocal microscopy, the LEA43 signal was also observed in dynamic foci structures along the ER with a diameter less than 1 µm (Figure 1A; Supplementary Figure S1B). In addition to the ER localization, LEA7, LEA9, LEA28, and LEA29 were also localized to nuclei as confirmed by DAPI staining (Figure 1B; Supplementary Figure S2).

For LEA42 and LEA48 lines, the fluorescent signal was detected in two organelle-like structures of approximately 1–1.5 µm in length with spherical shape and 0.5–1 µm in length with rod-like shape, suggesting plastidial and mitochondrial localization, respectively. Co-localization of the medium-sized structures with a fluorescent plastid reporter line confirmed LEA42 and LEA48 as plastidial proteins (Figure 1C (LEA42); Supplementary Figure S3A (LEA48)) [48], whereas the smaller structures overlapped with the Mitotracker signal indicating a mitochondrial localization (Figure 1D (LEA42); Supplementary Figure S3B (LEA48)).

### 3.3. LEA9 Forms Hydration Dependent Cytoplasmic Condensates in Embryos

**Figure 2:**
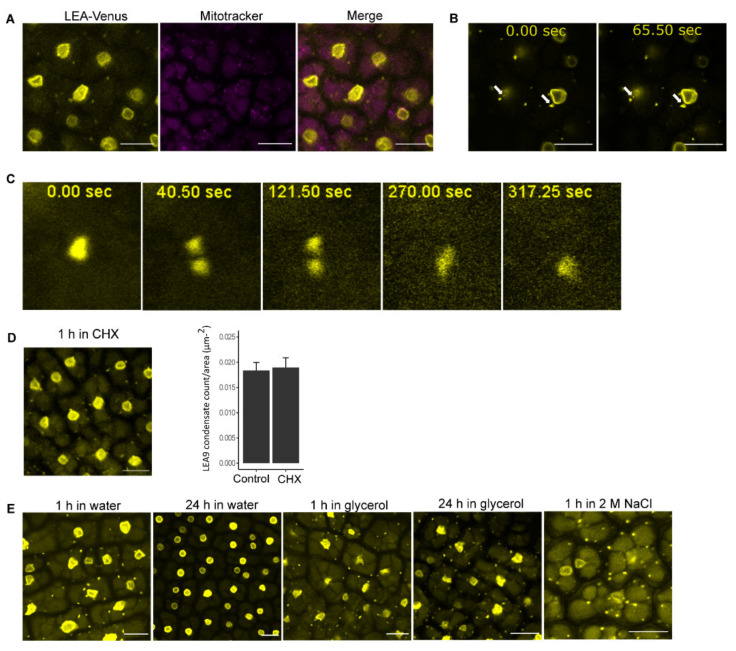
Cytoplasmic condensates of embryos of dry seeds from *proLEA9::LEA9:Venus* line. (**A**) Cytoplasmic condensates did not overlap with mitochondria by staining dissected embryos from seeds submerged in water for 1 h with Mitotracker. (**B**) Slight movement of cytoplasmic condensates as indicated by white arrows. (**C**) Division and fusion of cytoplasmic condensates. (**D**) Cytoplasmic condensate persistence after seeds were submerged in 1 mg/mL cycloheximide solution for 1 h. The bar plot shows counts of LEA9 condensates in seeds submerged in water or cycloheximide for 1 h (*n* = 9). (**E**) Observation of cytoplasmic condensates in dry seeds after imbibition in different conditions. Bars indicate 10 µm.

In embryo dissected from seeds imbibed in water for 1 h, LEA9 localized apart from nuclei in punctate structures in the cytoplasm (Figure 2). These LEA9 cytoplasmic structures showed an average diameter of approximately 1 µm. They were not localized in mitochondria (Figure 2A). Cytoplasmic LEA 9 condensates were mostly observed as static entities. However, some of these condensates showed dynamic behavior including dividing and merging (Figure 2B,C, Supplementary Video S1), which might suggest a liquid-like behavior.

Proteins that drive LLPS are usually IDPs or possess IDRs and most important show a multivalence character encoded by Short Linear amino acid Motifs (SLiMs) [51,52]. Using the MFDp2 protein disorder prediction tool [40], a 53.3% disorder content was predicted for LEA9, suggesting it to be an IDP. Additionally, we found several SLiMS scattered through the LEA9 sequence using The Eukaryotic Linear Motif (ELM) resource server (Supplementary Table S5). These results suggested that the observed LEA9 condensates might be the results of LLPS, which underlies formation of other well-known plant membrane-less organelles (MLOs) such as stress granules [53]. These cytoplasmic structures persisted in mature seeds of the *proLEA9::LEA9:Venus* line after treatment with cycloheximide, a chemical that prevents and dissolves the formation of stress granules [54]. The counts of LEA9 condensates in embryos dissected from seeds submerged in water or in cycloheximide for 1 h were not significantly different (Figure 2D). This suggested that they were unlikely to be stress granules, but most likely cytoplasmic condensates formed due to LLPS.

To examine the dynamics of cytoplasmic condensates, seeds from the *proLEA9::LEA9:Venus* line were subjected to different conditions before dissection (Figure 2). In addition to seeds imbibed in water for 1 h, these condensates were present when seeds were imbibed in glycerol or 2 M NaCl for 1 h before imaging (Figure 2E). When the incubation period in water and glycerol was expanded to 24 h, the condensates disappeared in water treated but not glycerol treated samples (Figure 2E), suggesting that their presence was influenced by the hydration status of the cells.

### 3.4. Cis-Acting Elements in the Promoter Regions of Seed-Expressed LEA_4 Genes

LEA gene expression is driven by various *cis*-acting elements in the respective promoter regions. The AtcisDB database in AGRIS was used to examine predicted motifs in the promoters of 11 seed-expressed LEA_4 proteins [42] (Supplementary Table S1A). LEA genes with the highest and lowest number of binding site motifs in their promoters were *LEA36* and *LEA43*, respectively. With the exception of *LEA9* all other LEA genes shared the most frequently found GATA promoter motif in their sequences, described as important for light-dependent and nitrate-dependent control of transcription [55] and stress responses [56]. RAV1-A and DPBF1&2 binding site motifs for ABI3/VP1 and bZIP and the ABA-responsive element (ABRE)-like binding site motif were also widely shared motifs among the investigated LEA promoters. On the other hand, some motifs were only found in promoters of certain LEA genes. For example, *LEA9* was the only LEA gene with a RAV1-B binding site motif while *LEA29* contained the most unique motifs as follows: Bellringer binding site 1, ATB2 binding site, L1-box promoter motif, and LS7 promoter element. High variation in *cis*-acting elements in the promoter regions of seed expressed LEA genes suggested different regulations of these genes. Genes of LEA proteins localized in the same subcellular structures shared some common promoter motifs (Supplementary Table S1B). No motif was, however, unique to the patterns of subcellular localization in this study.

### 3.5. Analysis of Seed-Expressed LEA_4 Proteins in Young Seedlings

Despite the decrease in gene expression and protein abundance of seed-expressed LEA_4 over time after germination [57], some LEA_4 proteins are reported to be detectable in young seedlings under the 35S promoter [30]. To assess expression patterns of 11 seed-expressed LEA_4 proteins under their native promoters, we analyzed 4-d-old seedlings expressing *proLEA_4::LEA_4:Venus* under control conditions using CLSM. LEA_4 proteins were expressed in various plant organs in young seedlings (Supplementary Figure S4). For LEA9 and LEA25, no Venus fluorescence signal was observed in young seedlings. Most LEA proteins were expressed in cotyledons, including epidermis and vascular bundles and in hypocotyls. Particularly, LEA7, LEA19, LEA28, and LEA29 were detected in roots. The expression pattern suggested that these proteins localized in regions adjacent to the phloem and root tips. More specifically, LEA29 was observed in the stele, most likely in the pericycle region, while LEA7, LEA19, and LEA28 were present in root caps. For LEA42 and LEA48, a slight signal was also detected in root caps. Only LEA36 was observed near to the zone of the stem cell niche.

### 3.6. LEA Proteins with the Same Subcellular Localization Form Homo- and Heterodimers

In this study, it was shown that LEA7, LEA28, and LEA29 (ER and nucleus), as well as LEA42 and LEA48 (mitochondria and plastids) shared the same subcellular localization in embryos dissected from dry seeds. To assess possible protein–protein interactions among LEA proteins, the formation of homo- and heterodimeric complexes was investigated using 2in1 ratiometric bimolecular fluorescence complementation (rBiFC) assays in leaves of *Nicotiana benthamiana*. Leaf discs from infiltrated leaves were analyzed under the confocal microscope.

**Figure 3:**
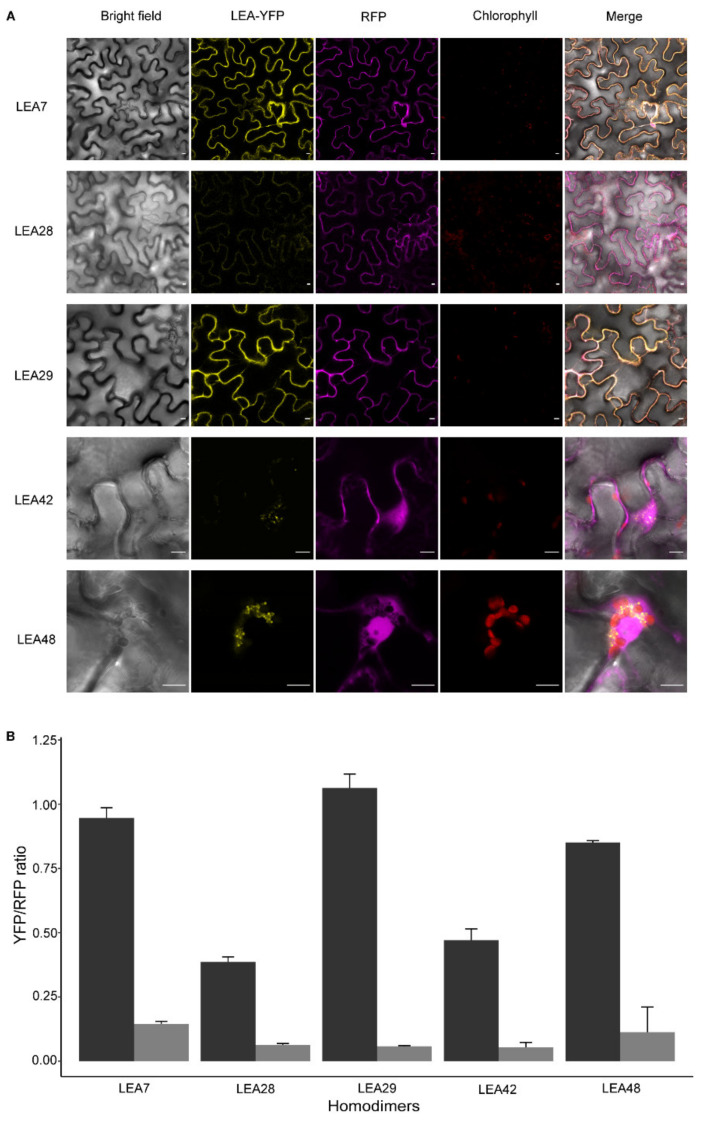
Homodimeric complex formation of LEA7, LEA28, LEA29, LEA42, and LEA48 in *N. benthamiana* leaves 4 d after infiltration. (**A**) Confocal microscope images. RFP channel is shown as reference marker. Bars indicate 10 µm. (**B**) Relative mean of LEA-YFP fluorescence normalized with the RFP reference marker (*n* = 15–25). Error bars show standard error. Black represents the interactions of each LEA protein while grey represents its negative control.

Homodimeric complex formation due to the reconstitution of the yellow fluorescent protein (YFP) was observed for LEA7, LEA29, and LEA48 but absent in LEA28 and LEA42 (Figure 3A). LEA7 and LEA29 homodimers were observed in the cytosol while for LEA48 the YFP signal was present as small droplet-like structures associated with the chloroplast. The formation of these homodimeric complexes was quantified using the relative YFP/RFP fluorescence ratio with a cut-off above 80% (Figure 3B).

**Figure 4:**
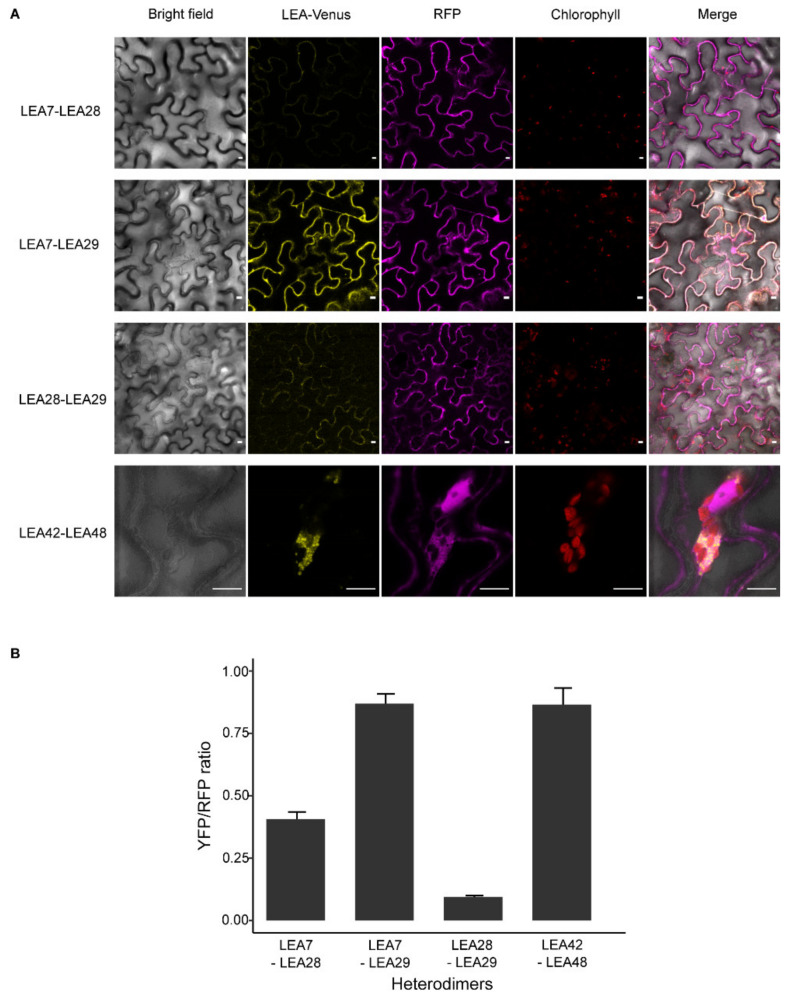
Heterodimeric complex formation of LEA7, LEA28, LEA29, LEA42, and LEA48 in *N. benthamiana* leaves 4 d after infiltration. (**A**) Confocal microscope images. RFP channel is shown as reference marker. Bars indicate 10 µm. (**B**) Relative mean of LEA-YFP fluorescence normalized with the RFP reference marker (*n* = 15-25). Error bars show standard error.

Assessing heterodimeric interactions between LEA7, LEA28, and LEA29 proteins revealed the formation of cytosolic heterodimeric complexes when nYFP-LEA29 was co-expressed with LEA7-cYFP (LEA7-LEA29) in epidermal cells (Figure 4A). However, an YFP signal was not observed during co-expression of LEA28/LEA7 and LEA28/LEA29. Similar to LEA48 homodimeric interactions, droplet-like structures were found when nYFP-LEA42 was co-expressed with LEA48- cYFP (LEA42-LEA48) (Figure 4A). The quantification of the YFP/RFP fluorescence of the complexes confirmed these interactions (Figure 4B).

**Figure 5:**
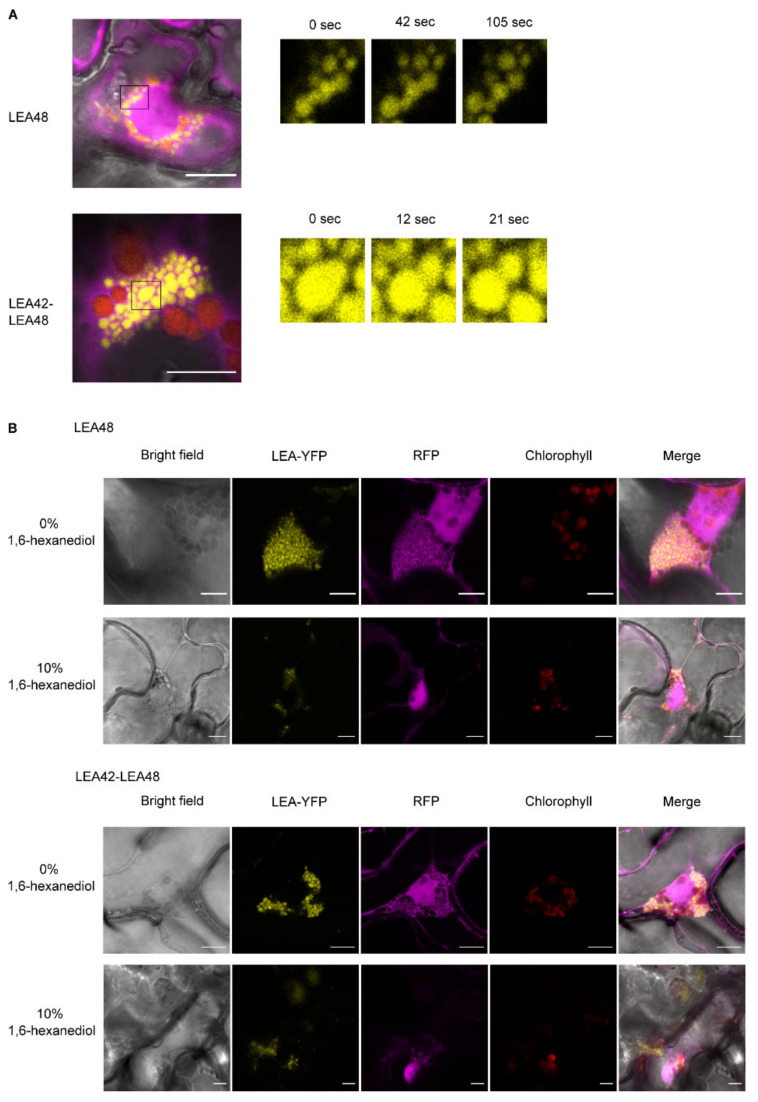
Liquid-liquid phase separation investigation of LEA48 homodimeric and LEA42-LEA48 heterodimeric complexes. (**A**) Separations of droplets. (**B**) Complexes after 0% or 10% 1,6-hexanediol treatment of *N. benthamiana* leaves 4 d after infiltration. Bars indicate 10 µm.

These droplet-like structures found for both LEA48 homodimeric and LEA42-LEA48 heterodimeric complexes were variable in size (from approximately 1–5 µm). Time-lapse confocal analysis of these structures revealed a liquid-like behavior as fusion and separation of droplets was detected for both LEA48 homodimers and LEA42-LEA48 heterodimers (Figure 5A).

The disorder content analysis of LEA42 and LEA48 using MFDp2 [40] predicted both proteins to be 100% disordered, suggesting the involvement of LLPS in complex formation. To assess whether LLPS was involved in the formation of these droplet-like structures 1,6-hexanediol, a chemical known to diffuse LLPS structures [58] was applied. Infiltrated leave samples were treated with 10% 1,6-hexanediol for 1 h followed by 10 min of vacuum-infiltration. This treatment was able to diffuse the droplet-like structures (Figure 5B). Therefore, it was hypothesized that the formation of LEA48 homodimeric and LEA42-LEA48 heterodimeric complexes was driven by LLPS.

## 4. Discussion

### 4.1. Subcellular Localization of Seed Expressed LEA_4 Proteins Differed Partly from Previous Studies

Plant LEA proteins have been reported to localize in a variety of subcellular structures [4]. Interestingly, most LEA groups showed a unique subcellular localization [30] apart from LEA_4 group. Different studies revealed their presence in the cytoplasm, plasma membrane, peroxisome, ER, nucleus, in chloroplasts and mitochondria [4,6,59]. Proteins of the groups LEA_1, LEA_2, LEA_5, LEA_6, dehydrin, and SMP have been reported to primarily localize in the cytosol or nucleus [6,30]. The AtM group, on the other hand, has been shown to localize within the secretory pathway, whereas LEA_3 members have been either localized in mitochondria or cytosol [30]. The ability of individual LEA proteins to stabilize specific macromolecules might promote their appearance in a multitude of subcellular localizations [23].

The eleven seed-expressed LEA_4 proteins, investigated in the present study in dissected seeds as their natural expression site, had a heterogeneous subcellular localization in seed embryos. They were localized in the ER (LEA19, LEA30, LEA25, LEA36, and LEA43), in nucleus and ER (LEA7, LEA28, and LEA29), in mitochondria and plastids (LEA42 and LEA48) and in the nucleus with simultaneous occurrence of cytoplasmic condensates (LEA9). These results were derived using for the first time a translational fusion with the Venus protein to the native promoter of LEA_4 proteins whereas findings of a previous publications were achieved using a 35S promoter fusion in protoplasts or seedlings. However, besides nuclear localization of some of these proteins, which matched with our findings, LEA7, LEA19, LEA25, LEA28, LEA29, and LEA36 were reported to localize in the cytoplasm of protoplasts [30], whereas these proteins localized to the ER in seed embryos. The differences between the two studies may arise from the different promoters and states of posttranslational modification of the proteins (see below). Another major difference was the subcellular localization of LEA9, which was previously reported in pexophagosomes of protoplast [30]. Here, we reported that LEA9 was localized in the nucleus and formed cytoplasmic liquid-like condensates under the native cellular context of the protein. LEA9 might passively leak into the nucleus as suggested for LEA proteins present in cytosol and nucleus, e.g., LEA7, LEA28, and LEA29, before [30]. The nucleus exclusion size was estimated to be around 60 kD [60] and LEA9 would still be a candidate with 52.7 kD [61] for possible leakage into the nucleus. On the other hand, a histidine-rich motif of the OpsDHN1 protein with a size of 40 kDa was described to regulate its nuclear localization arguing against a passive leakage [62]. Furthermore, different states of protein phosphorylation in different tissues might have caused different subcellular localization in seed embryos and protoplasts [63]. Enrichment regions of serine and threonine in IDPs and IDRs are more prone to phosphorylation events compared to ordered segments [14]. These regions might play prominent roles for posttranslational modifications necessary for defined interactions with partner proteins, thereby also influencing subcellular localization and functions [14].

Comparing the observed localizations with in silico predictions, six or five tools predicted the localization of the organelle localized LEA42 and LEA48 correctly, but only LOCALIZER suggested both organelles. The proposed localization to the nucleus from either BUSCA or Plant-mSubP (LEA7, LEA28, LEA29) or LOCALIZER (LEA9) was confirmed by the microscopic studies. None of the other bioinformatic tools suggested a localization to the nucleus for the respective LEA proteins. Only two tools predicted localization to the ER for LEA9, which was not confirmed by our studies. Prediction results for the majority of ER-localizing LEA proteins were cytoplasm and extracellular space from most of the prediction tools and these predictions overlapped with all ER-localized LEA proteins except LEA28, which was predicted to localize to nucleus and other organelles only. Finally, the tools BUSCA, Plant-mSubP, and LOCALIZER predicted the localization of five, five, and three LEA proteins out of eleven correctly in seeds, respectively. The conclusion, that prediction of protein localization can be improved using multiple predictions tools, was confirmed [30].

The investigation of the cellular localizations and interaction partners of LEA proteins can significantly improve our understanding of their precise biological functions [30]. However, further experimental in vivo approaches are needed to unravel the high complexity of the functional scenario of LEA proteins in connection to their subcellular localization [64].

The majority of seed expressed LEA_4 proteins were still detectable in seedlings in various organs, even that their abundance and the gene expression of the respective genes decreased during seed germination and seedling development [57]. LEA29 could even be detected in the phloem, where LEA proteins are only rarely found. In *Ricinus communis*, a metal-binding LEA-like protein was found to be involved in the transport of micronutrients [65,66] and the dehydrin HIRD11 was found in the phloem of *Brassica napus* (personal communication Julia Kehr, Hamburg University).

### 4.2. Homo- and Heterodimerization Was Found for LEA_4 Proteins with Same Subcellular Localization in Tobacco Leaves

Homo- and heterodimerization resulting in multiple protein complexes with varying target specifications are proposed to contribute to the fine-tuning of LEA protein driven stress responses [67]. Following this line, the dimerization of LEA_4 proteins with the same subcellular localization was investigated after transient expression in tobacco leaves. Homodimerization was found for LEA7, LEA29, and LEA48, but not LEA28 and LEA42, suggesting that LEA interactions might be protein specific. Cytosolic heterodimerization was shown for LEA7 and LEA29 and in addition, for LEA42 and LEA48, the only two LEA_4 proteins targeted to mitochondria as well as chloroplasts in this and another study [30].

The protein pairs LEA7, LEA29 and LEA42, LEA48 were previously described to be closely related with sequence identities above 45% [16]. This was only found for six LEA protein pairs encoded by homeologous genes resulting from duplication events [16]. Interestingly, LEA7 and LEA29, despite a sequence identity above 70%, differed highly in folding and function [61].

It was suggested before that, especially in seeds, the highly dehydrated status might favor conformation changes of LEA proteins resulting in formation of homo- or heterodimers, which then might be involved in the generation of the protective glassy state [12]. Further BiFC experiments are needed to determine LEA interactions in seeds.

LEA4-2 was previously described to form high molecular mass complexes when isolated from Arabidopsis cell extracts of stressed Arabidopsis plants [64]. For another class of LEA proteins, the dehydrins, homo- and heterodimerization, and even the generation of homotetramers [68] was shown, e.g., for COR85 from *Espinacea olaracea* [69], for dehydrin 2 (DHN2) from *Thellungiella salsuginea* [68], for WZY1-2 from wheat [70], for COR15 by size exclusion chromatography [71], and for COR47, ERD10, and RAB18 from Arabidopsis [67]. Formation of heterodimeric complexes was even shown between dehydrins from different species. Arabidopsis AtCOR47, AtERD10, and AtRAB18 dehydrins were able to interact with an acidic ortholog from *Opuntia streptacantha* [67]. These findings underline dimerization as a general property of LEA proteins possibly playing a role for their molecular function. Dimerization of the *O. streptacantha* SK3 dehydrin (DHN1) was shown to occur prior functioning, with K-segments together with the His rich motif and the presence of Zn^2+^ essential for the interaction [62]. Furthermore, LEA6 from common bean belonging to LEA group 6 proteins was shown to form dimers in vivo and a functional importance was suggested [72]. A LEA protein of the LEA group 7 from *Rosa chinensis* dimerized in leaves of Nicotiana [73]. As complex generation was seen for a wide range of LEA proteins from various groups, these interactions seem to be common for a high number of LEA proteins. Highly conserved sequences across the groups were proposed as important for formation of oligomers in stressed plants [64].

The formation of high order structures of homo- and heterodimers might result in the building of a more effective molecular shield. It was shown before that in vitro cryoprotection of lactate dehydrogenase activity was improved with an increased hydrodynamic dehydrin radius [74]. Furthermore, plants overexpressing two different dehydrins (RAB18 and COR47 or ERD10 and LTI30) from Arabidopsis were more freezing tolerant than control plants whereas transgenic lines expressing only one dehydrin were not different from the control despite comparable dehydrin levels [75]. LEA protein interaction was also suggested to prevent random protein degradation of these intrinsically disordered proteins [76,77].

Studying LEA protein localization and interactions contribute to the knowledge on their mechanisms of function and are first steps to understand the detailed stress involvement of the high variety of LEA proteins.

### 4.3. LEA9, the Homodimers of LEA48 and the Heterodimers of LEA42-LEA48 Undergo LLPS In Vivo

The involvement of LEA proteins in the formation of liquid-liquid phase separation (LLPS) or membrane-less organelles (MLOs) is an emerging and promising field that will shed light on the molecular mechanism that govern stress tolerance [78]. LEA proteins could potentially undergo LLPS to recruit their target proteins into droplets to protect them from the surrounding harsh environment [18]. In this study, the presence of cytoplasmic condensates with liquid-like and also static behavior in dissected embryos of LEA9 expressing lines was reported. Many proteins that initially form highly mobile liquid condensates become more viscoelastic and rigid over time, and eventually form a gel-like state that is unable to exchange its component molecules with the surrounding [79,80,81,82]. This transition could either be due to the entanglement of biopolymers or stronger association of proteins leading to fibril formation as reported for many protein condensates associated with neurological disorders such as FUS, TDP-43, Tau, and hnRNPA1 [79,80,82,83,84,85].

LEA9 condensates were modulated by the hydration state of the embryos as these condensates disappeared after a prolonged period of rehydration but were still visible in cotyledons and embryonic axis under stress conditions that evoke cellular dehydration, such as glycerol and osmotic stress treatments. Reversible LLPS results in a spontaneous de-mixing into a protein-rich and a protein-poor phase and depends on protein concentration and thresholds of external protein factors such as temperature, osmolarity, and pH [86,87]. These high dependence of condensate formation on variable factors might require unique regulation mechanisms [87]. Seeds are exposed to large environmental changes during imbibition and drying and might be places of frequent LLPS formation [18].

Additionally, droplet-like structures with dynamic behavior (fusion and separation patterns) were found for LEA48 homodimers and LEA42-LEA48 heterodimers after transient expression in tobacco leaves. Diffusion of these structures by 1,6-hexanediol underlined the hypothesis of their formation by LLPS. LLPS formation is often driven by IDPs or proteins with a high amount of IDRs, e.g., LEA proteins with high conformational flexibility as all membrane-less compartments incorporate high proportions of these proteins [53,88,89,90]. In addition, the ability of IDPs to scaffold protein–protein interactions due to its multivalent character have a crucial role to increase local protein concentration for LLPS formation [88].

Biomolecular condensates concentrate biomolecules [51] or buffer molecule concentration [91] and thereby contribute to dynamic biochemical compartmentation, modulation of spatiotemporal localization, and the regulation of cellular functions [53]. Formation of condensates by LLPS has been poorly investigated in plant systems, but was recently suggested as evolutionary ancient mechanism for organization within the cell [87]. In humans, where LLPS is more prevalently studied, abnormal pathological LLPS is constantly coming into focus being involved in cancer and neurodegenerative and cardiovascular diseases [92].

It was speculated before that, if LEA proteins are necessary for LLPS, then each LEA protein might recruit a specific target protein by protein–protein interaction [18]. These hypotheses would fit with our observation of LLPS for LEA48 homo- and LEA42-LEA48 heterodimers.

The study of condensates resulting from LLPS is complicated due to the complexity and flexibility of these structures [93]. Nevertheless, the occurrence of LLPS was demonstrated in vitro for many proteins forming condensates in vivo [87]. Further experiments using recombinant LEA9, LEA42, and LEA48 would help to determine LLPS properties of these proteins in vitro.

IDPs being highly sensitive to environmental changes are perfect candidates for fast LLPS transitions modulated by environmental inputs [92]. Non-stoichiometric assembled membrane-less structures are suggested despite a wide range of putative other function to dynamically adapt their composition to changing stress conditions allowing flexible stress responses [94].

Environmental sensitive phase separation was also suggested as a sensor of intra- and extracellular changes [95]. Reversible temperature-dependent phase separation was shown for Early flowering 3 (ELF3) and was suggested as temperature sensor for the circadian clock [96]. In seeds of Arabidopsis, a prion-like protein FLOE1 was identified to be involved in formation of LLPS during water uptake and in water stress sensing of the embryo [97]. The physiological role of LLPS was shown by the influence of the biophysical properties of FLOE1 condensates on the regulation of seed germination under unfavorable water conditions [97]. Stress-related LLPS generation was also suggested for the intrinsically disordered SR45 protein from Arabidopsis, accumulating in nuclear bodies dependent on temperature and protein phosphorylation, thereby promoting stress related splicing activity through concentration of the splicing components into LLPS [86]. In yeast, the stress granule protein Pub1 formed condensates in response to heat stress or starvation stopping the cell cycle [98]. Interestingly, different rates and physical properties were discovered for condensate dissolution, with starvation-induced condensates transformed into gel-like particles and more solid heat-induced ones requiring chaperones [98]. The lower movement of LEA9 condensates in comparison to high fusion and separation activities of LEA48 homodimers and LEA42-LEA48 heterodimers might either have been caused by the dense tissue structure in seeds or by rather a gel-like than a liquid-like structure as discovered for Pub1.

LEA proteins were suggested before to be candidates for phase separation to secure plant survival, e.g., during desiccation, when they are enriched in plants [87]. Environment-dependent transitions of LEA proteins from their disordered to an ordered state were suggested as sensing mechanism for LLPS formation as protection mechanism against irreversible protein aggregation [18]. LEA6 from the shrimp *Artemia franciscana* known to accumulate in anhydrobiotic embryos, contributed to improved desiccation tolerance by forming condensates for incorporation of desiccation-susceptible proteins dependent on their surface charge [78]. As no further literature in LEA protein condensates is available yet, further investigation of the specific properties of these LLPS driven condensates is necessary to increase the knowledge on the function of such structures in plants.

## 5. Conclusions

Subcellular localization of seed expressed LEA_4 group proteins driven by their native promoters varied partly from previous studies investigating protoplasts and seedlings. Several LEA proteins previously assigned to the cytoplasm were found in the endoplasmic reticulum. Bioinformatic tools for localization prediction predicted up to 45% of the selected proteins correctly and the usage of multiple tools for better results was suggested. Seed-expressed LEA proteins were, despite their deceasing abundance during germination and seedlings establishment, found in various organs of 4-d-old seedlings. Closely related proteins with the same subcellular localization (LEA7-LEA29 and LEA42-LEA48) were partly able to generate homo- and heterodimers after transient expression in tobacco leaves visualized by rBIFC. This dimerization might contribute to an improved molecular shielding of sensitive enzymes. LLPS, known to require IDPs as, e.g., LEA proteins, was discovered for LEA9 in Arabidopsis seeds and for homodimers of LEA48 and heterodimers of LEA42-LEA48 in tobacco leaves, possibly resulting in a concentration of the respective proteins. Condensates formed by LLPS could be important sensors of cellular changes and be crucial for stress responses.

This work sheds light on the specific subcellular LEA protein localization in seeds and contributes to the knowledge on dimerization of LEA proteins. Showing LLPS for three different LEA proteins or protein combinations proved this process as potential behavior of selected LEA proteins and underlined its importance in vivo.

## Figures and Tables

**Table 1:** Summary of the subcellular localization of seed-expressed LEA_4 proteins in embryos dissected from dry seeds. ER—Endoplasmic Reticulum. For ER-localized proteins, an additional cytoplasmic localization cannot not be ruled out completely.

LEA Protein	Subcellular Localization
LEA7	Nucleus, ER
LEA9	Nucleus, Cytoplasmic condensates
LEA19	ER
LEA25	ER
LEA28	Nucleus, ER
LEA29	Nucleus, ER
LEA30	ER
LEA36	ER
LEA42	Mitochondria, Chloroplasts
LEA43	ER
LEA48	Mitochondria, Chloroplasts

## Data Availability

Data are available on request.

## Supplementary Materials

The following are available online at https://www.mdpi.com/article/10.3390/biom11121770/s1. Supplementary Figure S1: Subcellular localization of seed-expressed LEA_4 proteins (LEA19, LEA28, LEA29, LEA30, LEA36) in ER in embryos of dry seeds. (A) proLEA_4::LEA_4:Venus lines were crossed with an ER marker line with CFP fluorescence (ER-ck). Bars indicate 10 µm. (B) The dynamics of LEA43 punctate along ER. Supplementary Figure S2: Subcellular localization of seed-expressed LEA_4 proteins (LEA9, LEA28, LEA29) in nuclei in embryos of dry seeds. Dissected embryos were stained with DAPI. Bars indicate 10 µm. Supplementary Figure S3: Subcellular localization of seed-expressed LEA_4 protein LEA48 in (A) plastids by crossing proLEA_4::LEA_4:Venus lines with a plastid marker line with CFP fluorescence (Pt-ck) and (B) mitochondria by staining dissected embryos with Mitotracker. Bars indicate 10 µm. Supplementary Figure S4: Localization of seed-expressed LEA_4 proteins different organs of 4-d-old seedling under control condition. If the organ is only shown with a bright field image, no fluorescence signal of Venus was detected. Red channel shows chlorophyll signal. White and black bars indicate 100 µm while blue bars indicate 10 µm. Supplementary Table S1: AGRIS promoter motif analysis. (A) Frequency of promoter motifs in each LEA proteins. AGRIS AtcisDB database was used for the motif identification. (B) Common promoter motifs among LEA proteins sharing the same subcellular localization. Supplementary Table S2: Length of native promoters and number of introns in LEA_4 group genes used for subcellular localization studies. Supplementary Table S3: Primer list. Supplementary Table S4: Localization prediction results of 11 seed-expressed LEA_4 proteins. The prediction showed either the subcellular structure(s) with the highest score of predictions or the results of the prediction with no scores. Empty spaces illustrate no prediction by that particular tool. CW—Cell Wall, ER—Endoplasmic Reticulum. Supplementary Table S5: Predicted Eucaryotic Linear Motifs (ELM) in LEA9 sequence. Supplementary Video S1: Division and fusion of cytoplasmic condensates of embryos of dry seeds from proLEA9::LEA9:Venus line.
