# Supplementary material for: Subcellular Localization of Seed-Expressed LEA_4 Proteins Reveals Liquid-Liquid Phase Separation for LEA9 and for LEA48 Homo- and LEA42-LEA48 Heterodimers
Source: Biomolecules. 2021 Nov 25;11(12):1770. doi: 10.3390/biom11121770 (PMC8698616; doi:10.3390/biom11121770)
Supplement: Supplementary file 1 [file biomolecules-11-01770-s001.zip › biomolecules-1417502-final-suppl/Supplementary Figures.pdf]

Ginsawaeng et al., 2021

Subcellular localization of seed-expressed LEA_4 proteins reveals liquid-liquid phase separation for LEA9 and for LEA48 homo- and LEA42-LEA48 heterodimers

Supplementary Figure S1

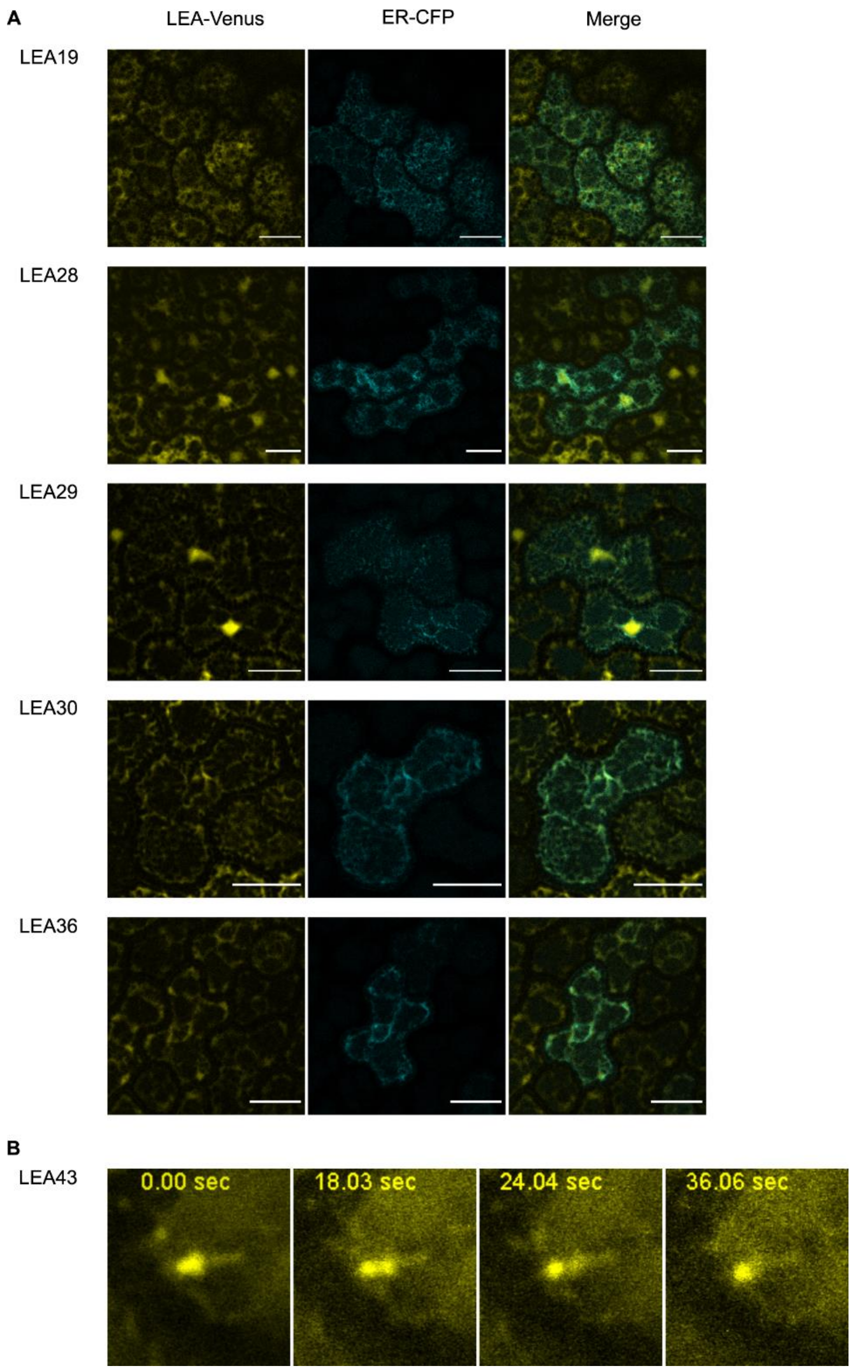

Supplementary Figure S1: Subcellular localization of seed-expressed LEA_4 proteins (LEA19, LEA28, LEA29, LEA30, LEA36) in ER in embryos of dry seeds. A) *pro*LEA_4::LEA_4:Venus lines were crossed with an ER marker line with CFP fluorescence (ER-ck). Bars indicate 10 µm. B) The dynamics of LEA43 punctate along ER.

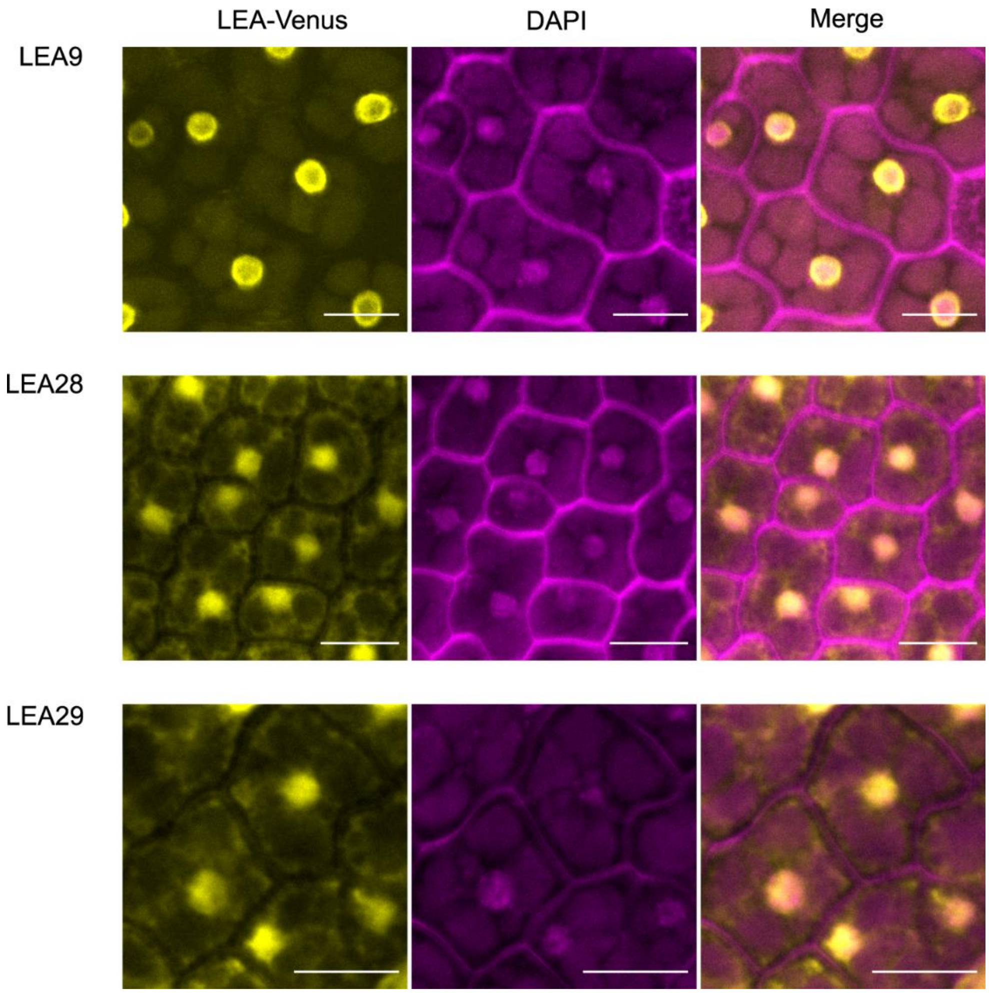

Supplementary Figure S2: Subcellular localization of seed-expressed LEA_4 proteins (LEA9, LEA28, LEA29) in nuclei in embryos of dry seeds. Dissected embryos were stained with DAPI. Bars indicate 10 μm.

Supplementary Figure S3

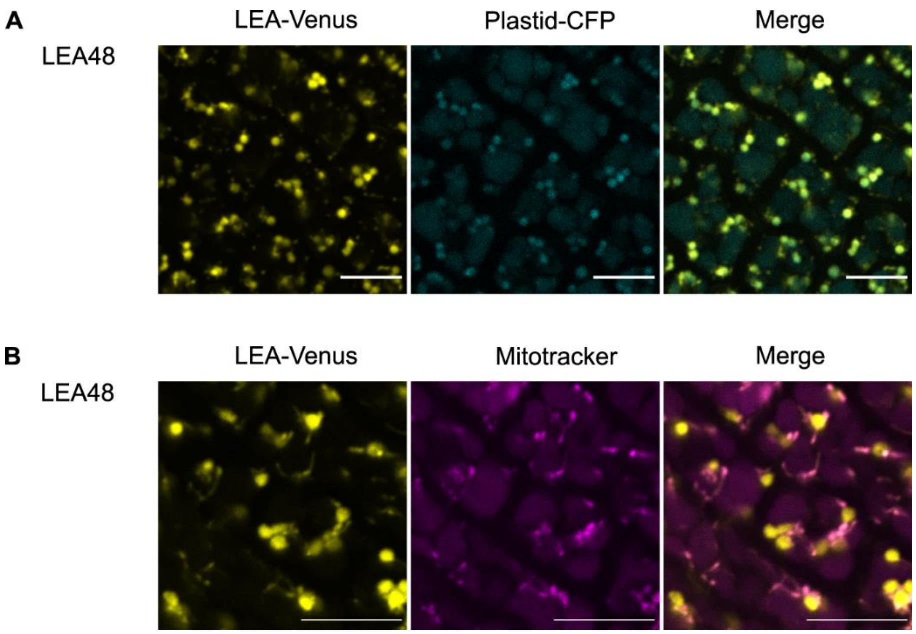

Supplementary Figure S3: Subcellular localization of seed-expressed LEA_4 protein LEA48 in A) plastids by crossing *pro*LEA_4::LEA_4:Venus lines with a plastid marker line with CFP fluorescence (Pt-ck) and B) mitochondria by staining dissected embryos with Mitotracker. Bars indicate 10 µm.

Supplementary Figure S4

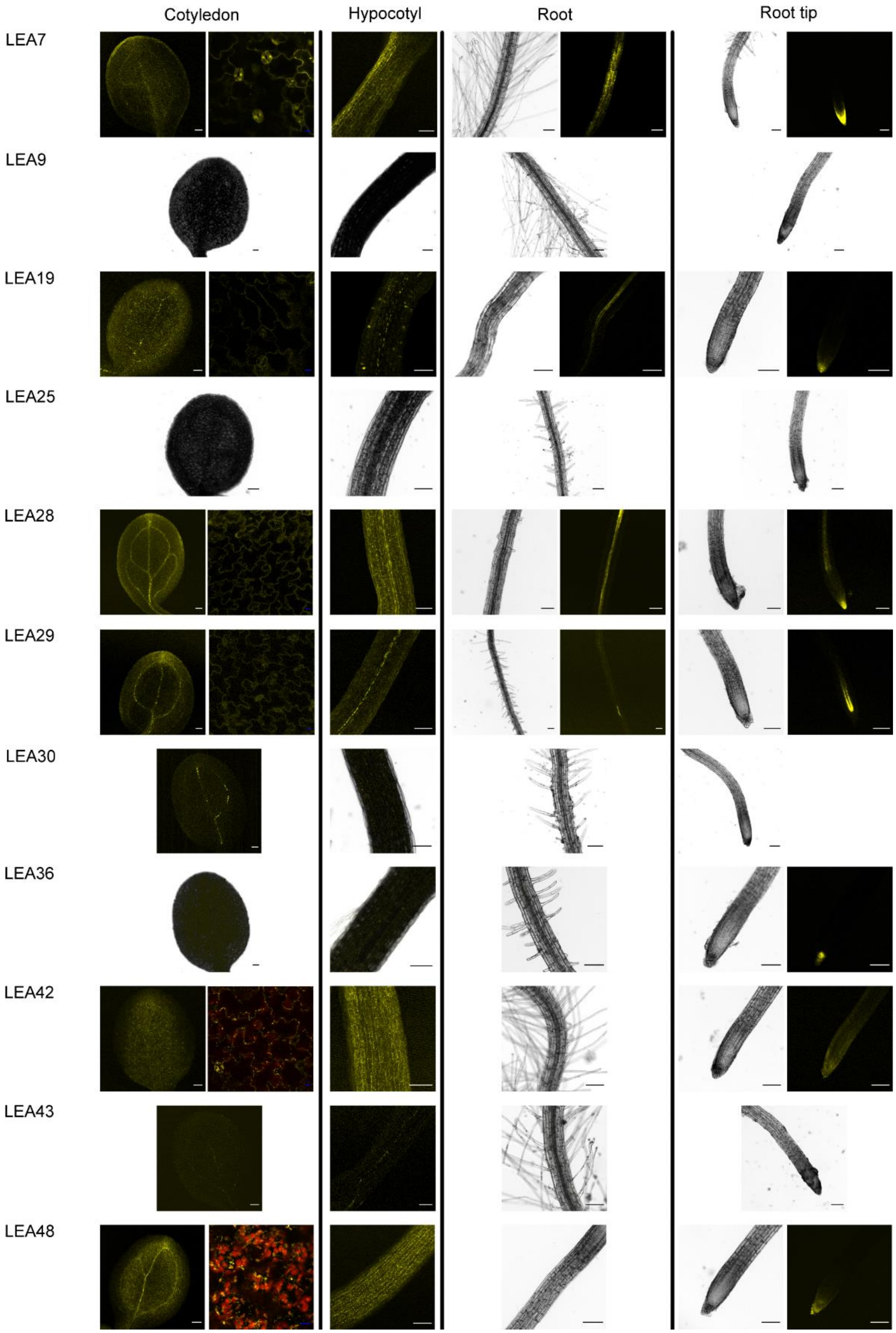

Supplementary Figure S4: Localization of seed-expressed LEA_4 proteins different organs of 4-d-old seedling under control condition. If the organ is only shown with a bright field image, no fluorescence signal of Venus was detected. Red channel shows chlorophyll signal. White and black bars indicate 100 μm while blue bars indicate 10 μm.
